# A Lightweight Breast Cancer Mass Classification Model Utilizing Simplified Swarm Optimization and Knowledge Distillation

**DOI:** 10.3390/bioengineering12060640

**Published:** 2025-06-11

**Authors:** Wei-Chang Yeh, Wei-Chung Shia, Yun-Ting Hsu, Chun-Hui Huang, Yong-Shiuan Lee

**Affiliations:** 1 Department of Industrial Engineering and Engineering Management, National Tsing Hua University, Hsinchu 300, Taiwan; yeh@ieee.org; 2 Department of Industrial and Systems Engineering, Chung Yuan Christian University, Taoyuan 320, Taiwan; 3 Laboratory of Molecular and Surgical Research, Department of Research, Changhua Christian Hospital, Changhua 500, Taiwan; 4 Department of Nursing, Chang Gung University, Taoyuan City 333, Taiwan; chhuang03@gmail.com; 5 Department of Applied Mathematics, Feng Chia University, Taichung City 407, Taiwan; yongslee@fcu.edu.tw

**Keywords:** lightweight breast cancer mass classification model, simplified swarm optimization, knowledge distillation, convolutional neural networks

## Abstract

In recent years, an increasing number of women worldwide have been affected by breast cancer. Early detection is crucial, as it is the only way to identify abnormalities at an early stage. However, most deep learning models developed for classifying breast cancer abnormalities tend to be large-scale and computationally intensive, often overlooking the constraints of cost and limited computational resources. This research addresses these challenges by utilizing the CBIS-DDSM dataset and introducing a novel concatenated classification architecture and a two-stage strategy to develop an optimized, lightweight model for breast mass abnormality classification. Through data augmentation and image preprocessing, the proposed model demonstrates a superior performance compared to standalone CNN and DNN models. The two-stage strategy involves first constructing a compact model using knowledge distillation and then refining its structure with a heuristic approach known as Simplified Swarm Optimization (SSO). The experimental results confirm that knowledge distillation significantly enhances the model’s performance. Furthermore, by applying SSO’s full-variable update mechanism, the final model—SSO-Concatenated NASNetMobile (SSO-CNNM)—achieves outstanding performance metrics. It attains a compression rate of 96.17%, along with accuracy, precision, recall, and AUC scores of 96.47%, 97.4%, 94.94%, and 98.23%, respectively, outperforming other existing methods.

## 1. Introduction

Breast cancer is one of the most common types of cancer among women worldwide, ranking as the second most prevalent cancer in women globally, following lung cancer [1]. According to the literature, early detection and treatment can increase the cure rate of breast cancer from 40% to 90% [2]. Among breast cancer screening tools, mammography is the most widely used method [3,4]. With advancements in machine learning and deep learning, these technologies have also been applied to computer-aided diagnosis (CAD) systems [5] to perform classification tasks on mammographic images. Mammographic image classification tasks can generally be divided into four categories [6]—breast density classification, breast asymmetry classification, breast mass classification, and breast calcification classification. Among these, breast mass and breast calcification are regarded by radiologists as early indicators of breast cancer [7]. Therefore, most studies focus on breast mass classification and breast calcification classification, often utilizing publicly available mammographic image datasets for model training. Due to the limited number of images in commonly used mammographic datasets, researchers employ data augmentation techniques to increase the amount of training data in order to prevent model overfitting [8] and to enhance the model’s generalization ability. In addition, transfer learning (TL) [9] is frequently used to improve model performance. Transfer learning is a machine learning approach where knowledge from a source domain is transferred to a target domain, enabling a pretrained model to solve problems in a new domain without the need for training from scratch. This experience-based learning strategy allows models to learn more quickly and efficiently.

Research on abnormality classification in mammographic images can be broadly categorized into two types based on the training data used—Region of Interest (ROI) images and full-field images [10,11]. Full-field mammograms present significant challenges for machine learning due to the extremely small tumor-to-image ratio (approximately 100 × 100 pixels versus 5900 × 4700 pixels) [12,13] and substantial noise from non-breast tissues and backgrounds. In contrast, ROI (Region of Interest) images, which are cropped to suspicious lesion areas (average 371 × 368 pixels), offer three key advantages—minimal information loss during resizing, reduced distortion due to consistent aspect ratios, and the elimination of irrelevant information for more effective CNN model training. Therefore, ROI images are more suitable for breast cancer detection applications.

Mammographic abnormality classification has evolved through distinct methodological approaches, each with inherent trade-offs between performance and practicality. Early studies relied on traditional machine learning techniques that manually extracted statistical features [14,15,16] followed by classification using algorithms such as Support Vector Machines (SVM) [17], Random Forests [18], and Extreme Gradient Boosting (XGBoost) [19]. While these approaches offered straightforward implementation, they suffered from complex preprocessing procedures and limited generalization ability.

Traditional machine learning methods still play an important role in breast cancer classification in certain scenarios, such as when the dataset is limited or when model interpretability must be preserved in clinical applications. In particular, existing studies using cone-beam CT images [19], dynamic contrast-enhanced MRI (DCE-MRI) [20], and contrast-enhanced mammography (CEM) [21] show that models like back propagation neural networks and LASSO regression with high-dimensional radiomic features achieve satisfying diagnostic accuracy while maintaining interpretability and compatibility with standard imaging workflows.

The emergence of deep learning has transformed medical imaging through convolutional neural networks (CNNs) [6], which automatically extract features via specialized layers including convolutional layers, batch normalization layers [22], and global average pooling layers [23], eliminating the need for manual feature engineering. Recent developments have favored large-scale ensemble models that combine multiple pretrained networks or multi-view feature fusion approaches, achieving a superior performance by learning from diverse data sources [24,25]. Research has demonstrated a proportional relationship between model accuracy and parameter count [26,27], driving the trend toward increasingly complex architectures.

However, these high-capacity models demand substantial memory, powerful computational units, and significant time resources, creating practical deployment challenges [28,29]. The optimization of hyperparameters further complicates their implementation, highlighting the ongoing tension between achieving maximum accuracy and maintaining computational feasibility in clinical settings.

Recent advances in deep learning have introduced lightweight and high-performing architectures for medical imaging tasks, including mammography. EfficientNet-lite, a computationally efficient variant of EfficientNet, has shown a strong performance with a reduced model size and inference time, making it suitable for deployment in resource-constrained clinical settings [30]. Meanwhile, Vision Transformers (ViTs) offer enhanced global feature modeling and have demonstrated promising results in capturing subtle patterns in breast tissue, though they typically require large training datasets and are more computationally intensive [31].

Hybrid models combining CNNs with transformer blocks aim to leverage the strengths of both local and global representations, improving classification accuracy in mammography tasks [32,33,34]. However, their architectural complexity may hinder their real-time deployment. These frameworks continue to evolve, balancing performance, interpretability, and scalability for clinical integration.

Traditionally, hyperparameter tuning has been conducted manually based on empirical rules, a process that is often tedious and inefficient. Consequently, various automatic hyperparameter optimization methods have been proposed, including Grid Search (GS) [35], Bayesian Optimization (BO) [36], Randomized Search (RS) [37], Genetic Algorithm (GA) [38], Particle Swarm Optimization (PSO) [39], and Simplified Swarm Optimization (SSO) [40].

Heuristic algorithms such as GA and Swarm Optimization (SO) have been widely applied for fine-tuning CNN hyperparameters. Among these, SSO is particularly noted for its simple update mechanism and ease of implementation. Numerous studies have demonstrated that SSO can efficiently find optimal solutions in optimization problems [41,42,43].

Current mammographic image classification research predominantly emphasizes accuracy improvements while overlooking computational efficiency and real-world deployment constraints. Existing high-capacity models, though achieving a superior performance, demand substantial computational resources that limit their clinical applicability. Additionally, most studies utilize single data modalities, potentially missing valuable complementary information from heterogeneous sources. To address these limitations, this study proposes a novel lightweight concatenated classification model that integrates both imaging and tabular data while maintaining computational efficiency through model compression techniques.

The key contributions of this study are as follows:

1. Multi-modal Data Integration: Development of a concatenated classification model that combines imaging and tabular data features, enabling the model to leverage complementary information from heterogeneous sources for enhanced breast tumor classification performance.
2. Lightweight Model Architecture: Implementation of knowledge distillation techniques to create a computationally efficient model that significantly reduces parameter count and computational costs while preserving classification accuracy.
3. Heuristic Optimization: Application of the Simplified Swarm Optimization (SSO) algorithm to fine-tune model hyperparameters, ensuring optimal performance through global optimization while avoiding local minima constraints.

## 2. Preliminaries

This section provides an overview of knowledge distillation and SSO, including their foundational ideas and practical applications.

### 2.1. Overview of Knowledge Distillation

Model resource requirements differ significantly between the training and deployment phases. While training utilizes complex architectures on large datasets for enhanced generalization, deployment must consider hardware limitations and latency constraints in clinical environments. Knowledge distillation addresses this challenge by compressing trained models while preserving performance.

Introduced by Caruana et al. [44] in 2006 and refined by Geoffrey et al. [45] in 2015, knowledge distillation transfers knowledge from a complex “teacher” model to a smaller “student” model through a two-phase training process. The student model learns from both soft labels (vs. teacher outputs) and hard labels (vs. ground truth) using the following combined loss function:

(1) $$L=\alpha L_{soft}+(1-\alpha )L_{hard}$$

Traditional hard labels use standard softmax, as follows:

(2) $$q_{i}=\frac{exp\left(z_{i}\right)}{\sum_{i}exp\left(z_{i}\right)}$$

Knowledge distillation employs temperature-adjusted softmax for informative soft labels, as follows:

(3) $$q_{i}=\frac{exp\left(z_{i}/T\right)}{\sum_{i}exp\left(z_{i}/T\right)}$$

The distillation and student losses are calculated as follows:

(4) $$L_{soft}=-\sum_{i}^{N}p_{i}^{T}\log\left(q_{i}^{T}\right),\;where{\;p}_{i}^{T}=\frac{\exp\left(v_{i}/T\right)}{\sum_{k}^{N}\exp\left(v_{k}/T\right)},q_{i}^{T}=\frac{\exp\left(z_{i}/T\right)}{\sum_{k}^{N}\exp\left(z_{k}/T\right)}$$

(5) $$L_{hard}=-\sum_{i}^{N}c_{i}\log\left(q_{i}^{1}\right),where\;q_{i}^{1}=\frac{\exp\left(z_{i}\right)}{\sum_{k}^{N}\exp\left(z_{k}\right)}$$

where *N* denotes the total number of labels; *v_i_* and *z_i_* represent the logits from the teacher model and the student model, respectively; $p_{i}^{T}$ and ${\;q}_{i}^{T}$ denote the softmax outputs for class iii from the teacher and student models under the temperature hyperparameter *T*; and *c_i_* represents the ground truth label for class *i*, where *c_i_* ∈{0,1}.

Despite proven effectiveness across various domains, knowledge distillation remains underexplored in breast cancer classification, motivating this study’s application to lightweight model development.

### 2.2. Simplified Swarm Optimization

CNN hyperparameter optimization represents an NP-hard problem that is traditionally addressed through guesswork or heuristic rules, often failing to achieve optimal solutions [46]. Swarm intelligence-based heuristic algorithms offer effective alternatives for finding near-optimal solutions within reasonable timeframes.

Simplified Swarm Optimization (SSO) [47], proposed by Yeh in 2009, encodes each particle as a positive integer representing feasible system structures. The algorithm employs a step function for solution updates, governed by hyperparameters *C_g_*, *C_p_*, and *C_w_* with the constraint 0 < *C_g_* < *C_p_* < *C_w_* < 1, as follows:

(6) $$x_{i,j}^{t+1}=\left\{\begin{array}{c} x_{i,j}^{t}\;if\;\rho_{\left[0,1\right]}\in \left[0,C_{g})\right.\\ p_{i,j}^{t}\;if\;\rho_{\left[0,1\right]}\in \left[C_{g},C_{p})\right.\\ g_{j}\;if\;\rho_{\left[0,1\right]}\in \left[C_{p},C_{w})\right.\\ x\;if\;\rho_{\left[0,1\right]}\in \left[C_{w},1)\right. \end{array}\right.$$

The update mechanism involves four probabilistic cases—maintaining current value, adopting personal best, selecting global best, or random replacement. The random assignment component enhances solution diversity, preventing local optima entrapment and facilitating convergence toward global optima.

SSO utilizes orthogonal arrays for optimal parameter combination identification [48] and has demonstrated effectiveness across diverse applications including breast cancer classification [49], neural network training [50], and time series forecasting [50], proving its capability in handling discrete variable optimization problems.

## 3. Materials and Methods

This section introduces the CBIS-DDSM dataset in Section 3.1, followed by a description of the data preprocessing procedures in Section 3.2. Section 3.3 provides a detailed explanation of the model architecture and the two-stage methodological framework. Section 3.4 describes the four model evaluation metrics. Finally, Section 3.5 explains the solution encoding/decoding strategies and the update mechanism of the Simplified Swarm Optimization algorithm.

### 3.1. Dataset Description

This study utilizes the CBIS-DDSM (Curated Breast Imaging Subset of DDSM) dataset (CA, USA) as the data source for the research on breast tumor abnormality classification. The CBIS-DDSM is an updated and standardized version of the DDSM (Digital Database for Screening Mammography) dataset. As a curated and professionally managed dataset, it contains decompressed mammographic images converted to DICOM format, updated ROI annotations and bounding boxes, and the corresponding pathological diagnoses for the images.

The CBIS-DDSM dataset contains 1696 samples of tumor abnormalities. Each sample includes three types of imaging data. The imaging data consist of three types—the full mammographic image, the ROI mask, and the cropped ROI image. The corresponding tabular data, as summarized in Table 1, record detailed information about the tumor abnormalities. The tabular data include the following fields: patient ID, breast density, laterality (left or right breast), image view, abnormality ID, abnormality type, mass shape, mass margins, BI-RADS assessment, pathology, subtlety, image file path, cropped image file path, and ROI mask file path.

Breast density is categorized into four levels (1–4). Laterality distinguishes between the left and right breasts. Image views include craniocaudal (CC) and mediolateral oblique (MLO) perspectives. Abnormality IDs range from 1 to 7. The abnormality type in this study focuses on tumor abnormalities. Mass shape is classified into 20 types, while mass margins are categorized into 19 types. BI-RADS assessments range from 0 to 5. Subtlety is rated on a scale from 1 to 5. Pathological diagnoses are categorized into three classes—benign, benign without callback, and malignant. Since the number of cases labeled as “benign without callback” is relatively small, this study treats them as “benign” to better emphasize the binary classification between benign and malignant cases.

This study uses the 1696 ROI images and corresponding tabular data provided by the CBIS-DDSM dataset as an input for the model. The dataset is divided into training, validation, and test sets, with 80% allocated for training, 10% for validation, and 10% for testing. Additionally, to improve the model’s training performance and ensure satisfactory results on the test set, the benign-to-malignant ratio in each of the three datasets is adjusted to closely match the ratio in the original dataset. This ensures that the distribution of benign and malignant cases in each subset is consistent with the original dataset, as shown in Table 2.

### 3.2. Data Preprocessing

The purpose of data preprocessing is to reduce noise and enable the model to train more effectively. Therefore, the following preprocessing steps are applied to both the tabular data and ROI image data.

#### 3.2.1. The Tabular Data

In this study, six important columns are extracted from the original tabular data as input features (X) and class labels (Y) for the model. Five columns are used as input features (X), which include breast density, tumor shape, tumor margins, BI-RADS assessment, and subtlety. The remaining column serves as the class label (Y), which is the pathological diagnosis. Since tumor shape, tumor margins, and pathological diagnosis are categorical data, these categorical variables must be converted into numerical data that the model can process. For tumor shape and tumor margins, one-hot encoding is applied to convert each type in these columns into multiple independent columns. For example, since tumor shape consists of 20 types, the number of columns will increase from 1 to 20 independent columns. Similarly, tumor margins consist of 19 types, so the number of columns will expand to 19 independent columns. Each cell in these columns is populated with binary values (1 or 0), where 1 indicates the presence of the respective type and 0 indicates its absence. For missing data in the tumor shape (4 missing cells) and tumor margins (60 missing cells) columns, an unknown (UNKNOWN) type is introduced to fill in these gaps. Consequently, the tumor shape column will have 21 independent columns, and the tumor margins column will have 20 independent columns. As for the pathological diagnosis, which is the target variable to be predicted by the model, benign and malignant cases are replaced by 0 and 1, respectively.

#### 3.2.2. ROI Image Data

Since the aspect ratio and size of the ROI images do not match the input format required by the CNN model, the images will be resized to ensure they are compatible with the model’s input format, while balancing efficiency and performance. To avoid issues such as vanishing or exploding gradients during parameter optimization with gradient descent, which are caused by significant differences in pixel scales across different images, the pixel values of the images will be standardized. This ensures that the influence of different image features on the parameters is consistent. Furthermore, because mammographic images have limited resolution and are prone to artifacts, three preprocessing steps will be applied to improve image classification accuracy. These steps, in order, are the removal of white borders, non-local mean filtering, and contrast-limited adaptive histogram equalization (CLAHE). The following sections provide a detailed explanation of these methods.

1. Removal of White Borders

**Figure 1:**
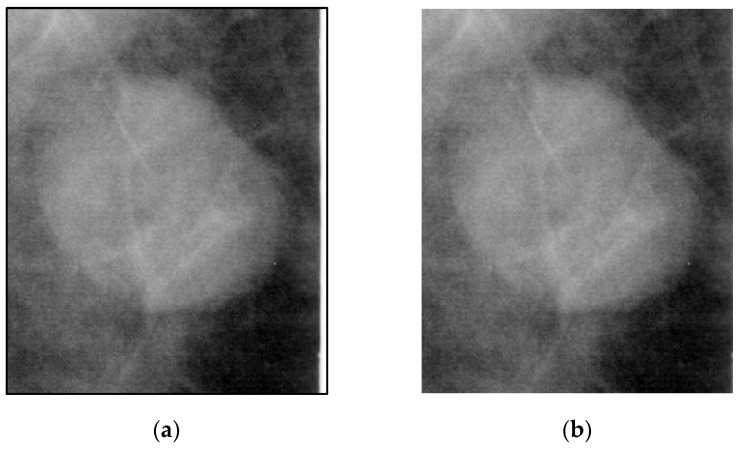
Comparison of an ROI image with and without white borders. (**a**) White borders in the ROI image (image with black frame). (**b**) ROI image after the removal of white borders.

Some ROI images contain white borders, as shown in Figure 1. These white borders may be artifacts introduced during the mammographic imaging process and act as noise for the model, potentially impacting its training performance. Therefore, the white borders are removed to reduce interference with the model.

2. Non-Local Mean Denoising Algorithm

**Figure 2:**
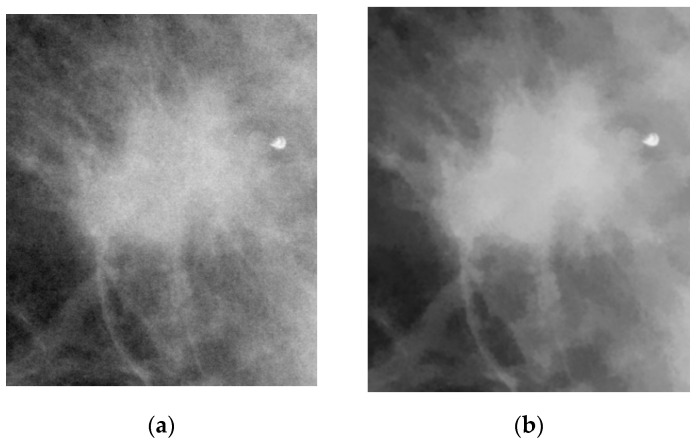
Comparison of ROI images before and after applying the NLM algorithm. (**a**) Original ROI image. (**b**) ROI image after applying the NLM algorithm.

Non-local mean (NLM) denoising is an image denoising algorithm that compared to local mean denoising, which only uses the neighboring points around each target pixel to smooth the image and remove noise, defines a block around each target pixel and assigns weights based on the similarity of the surrounding blocks across the entire image. The current pixel’s estimated value is obtained by taking the weighted average of pixels that have a similar neighborhood structure. The NLM algorithm improves image clarity while preserving more details. Figure 2 shows a comparison of the image before and after applying the NLM algorithm.

3. Contrast-Limited Adaptive Histogram Equalization—CLAHE

**Figure 3:**
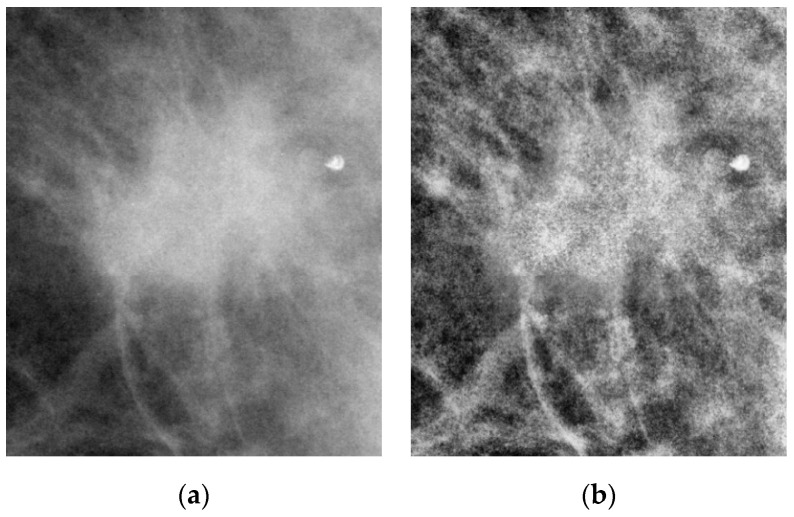
Comparison of ROI images before and after applying CLAHE. (**a**) Original ROI image. (**b**) ROI image after applying CLAHE.

CLAHE divides the image into multiple regions (e.g., 8 × 8), and histogram equalization is applied to each region individually to enhance local contrast. The contrast of the entire image can be controlled by setting a threshold for the maximum pixel value within each region. Figure 3 shows a comparison of the image before and after applying CLAHE.

### 3.3. Methodology Framework

**Figure 4:**
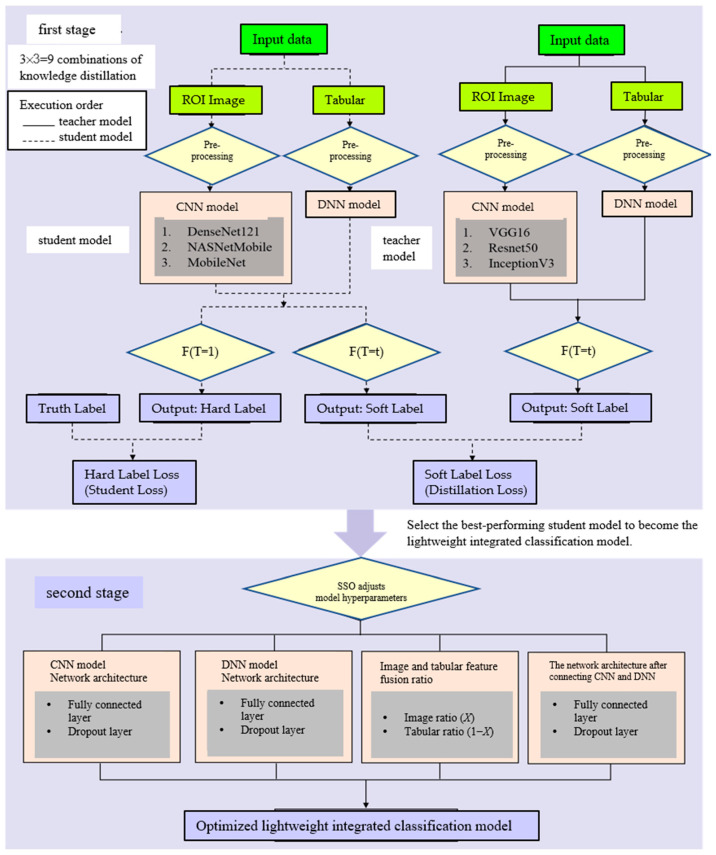
A two-stage framework diagram illustrating the model architecture.

The preprocessed image and tabular data will serve as an input for the integrated classification model proposed in this study, followed by a two-stage execution process, as shown in Figure 4.

In the first stage, knowledge distillation will be conducted, where the teacher model is a large-scale integrated classification model and the student model is a small-scale integrated classification model. Through knowledge distillation, the student model is trained to become a lightweight integrated classification model with a performance comparable to that of the teacher model. During the distillation process, the teacher model is first trained; once the training is complete, the student model is then trained via knowledge distillation. By computing the distillation loss function and the student loss function, the student model learns from both the teacher model and the true labels, thereby enhancing its generalization ability. A total of nine combinations of knowledge distillation will be performed during this stage. Ultimately, the student model that achieves the highest accuracy while maintaining a performance close to that of the teacher model will be selected as the lightweight integrated classification model and will serve as the target for optimization in the second stage using SSO.

In the second stage, the structure of the lightweight integrated classification model will be optimized using SSO. The optimization primarily involves adjusting the fusion ratio between image features and tabular features, as well as tuning hyperparameters at three points in the model architecture—the CNN model, the DNN model, and the network structure connecting the CNN and DNN models. For each of these three components, two hyperparameters will be adjusted—the number of neurons in the fully connected layers and the dropout rate controlling the probability of randomly deactivating neurons. In total, seven hyperparameters will be optimized to construct an optimized lightweight integrated classification model. The following sections will provide a detailed explanation of the architectures of the integrated classification model and the teacher–student models.

#### 3.3.1. Integrated Classification Model

The integrated classification model is primarily composed of two submodels—a CNN model and a DNN model.

The CNN model is primarily used to train on image data. To accelerate training time and enhance performance, the pretrained model weights provided by the TensorFlow Keras API, which were trained on the ImageNet dataset, are utilized. Through transfer learning, the pretrained weights are used to initialize the CNN model within the integrated classification model, enabling it to train on and extract features from mammography images. Six classical pretrained CNN models—VGG16, ResNet50, InceptionV3, DenseNet121, NASNetMobile, and MobileNet—are employed for the CNN component of the integrated classification model.

Since the final classification layers of these models were originally designed for the 1000-class prediction task on the ImageNet dataset, this study modifies these layers to fit the binary classification task of breast tumor abnormality detection by setting include_top = False in the TensorFlow Keras API. The modified network architecture sequentially passes through a global average pooling layer, a fully connected layer, and a dropout layer.

Additionally, the input dimensions of the ROI images are resized according to Table 3. To balance model performance and computational efficiency, the input dimensions for both the images and models are uniformly resized to (75, 75, 3).

The DNN model is primarily used to train on tabular data. The model architecture consists of a fully connected layer and a dropout layer. After preprocessing, the tabular data consist of 44 features, which are represented as a 44-dimensional feature vector and are input into the next fully connected layer. The output is then determined by the corresponding number of neurons in that layer, followed by a dropout layer where neurons are randomly deactivated to prevent overfitting.

**Figure 5:**
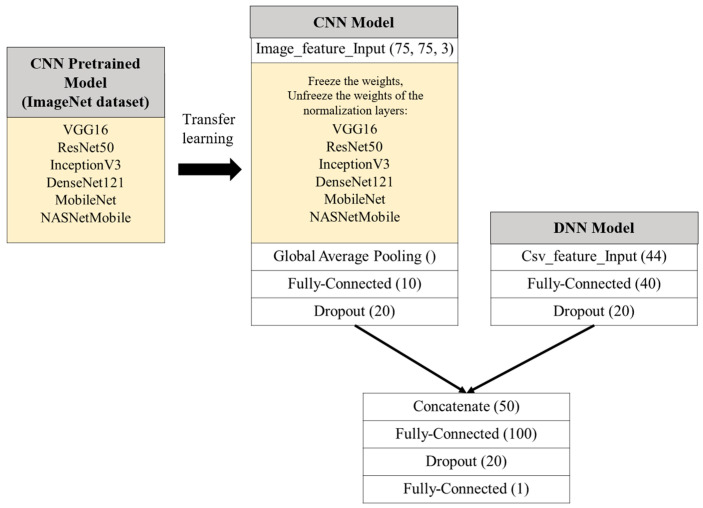
The integrated classification model architecture proposed in this study.

The architecture of the integrated classification model proposed in this study is shown in Figure 5. After the CNN model passes through the pretrained model, it sequentially passes through a global average pooling layer, a fully connected layer, and a dropout layer, producing a one-dimensional feature vector. This vector is then concatenated with the one-dimensional feature vector from the DNN model, which has passed through its own fully connected and dropout layers. In the CNN model, the six pretrained CNN models, which were trained on the ImageNet dataset, will be utilized. To effectively leverage the pretrained weights and harness the potential of transfer learning, all parameters except for the normalization layers will be frozen. The weights of the normalization layers will be unfrozen, allowing the model to recalculate the mean and variance for each batch based on mammography images, thereby training the model’s parameter weights. These adjusted weights will serve as the initialization weights.

Finally, the concatenated feature vector is passed through a fully connected layer and a dropout layer before outputting the model’s prediction, i.e., 0 (benign) or 1 (malignant). Based on the different CNN pretrained models, six distinct integrated classification models are created, namely Concatenated VGG16 (ConVGG16), Concatenated ResNet50 (ConResNet50), Concatenated InceptionV3 (ConInceptionV3), Concatenated DenseNet121 (ConDenseNet121), Concatenated NASNetMobile (ConNASNetMobile), and Concatenated MobileNet (ConMobileNet).

#### 3.3.2. Lightweight Integrated Classification Model

The number of model parameters affects the model’s predictive capability. When there are sufficient data, the larger the model, the better its predictive performance. However, a larger model comes with a significant increase in computational cost due to the larger number of parameters. For example, in the integrated classification model proposed in this study, the number of parameters in the VGG16 model for image inputs of dimension (75, 75, 3) is in the order of tens of millions. To reduce the number of parameters and minimize the demand for computational resources, this study utilizes knowledge distillation to construct a lightweight integrated classification model.

Since the bottleneck of the integrated classification model primarily occurs in the CNN model handling the images, the model size and parameter count shown in Table 4 will serve as the basis for selecting the teacher and student models. Finally, larger models with more parameters, such as VGG16, ResNet50, and InceptionV3, will be used as the teacher model CNN architectures, while smaller models with fewer parameters, such as DenseNet121, NASNetMobile, and MobileNet, will serve as the student model CNN architectures.

#### 3.3.3. Knowledge Distillation

**Figure 6:**
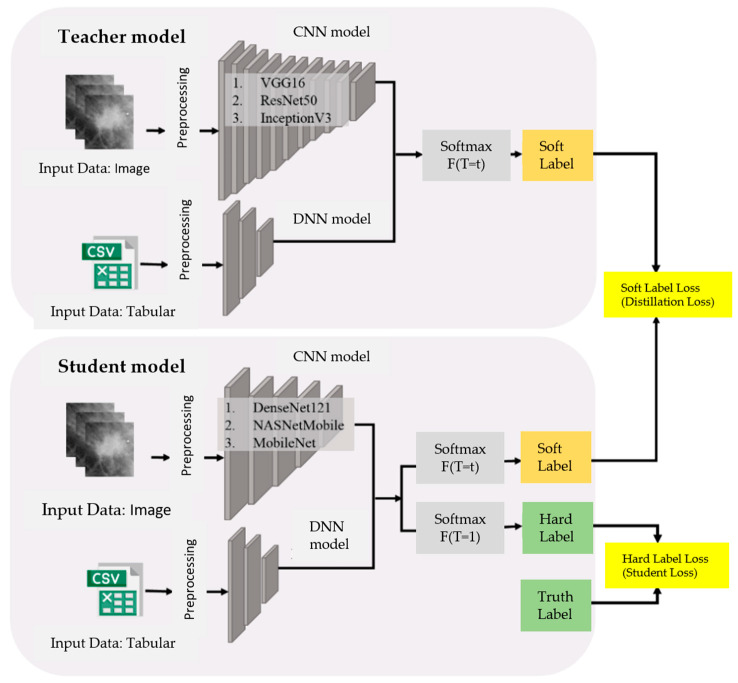
Knowledge distillation process.

Based on the number of parameters and the model size of the six CNN pretrained models mentioned above, they can be primarily divided into three teacher models—ConVGG16, ConResNet50, and ConInceptionV3—and three student models—ConDenseNet121, ConNASNetMobile, and ConMobileNet. The teacher models and student models will be paired in a one-to-one manner, resulting in a total of nine knowledge distillation pairs. Through knowledge distillation, the student models can learn the generalization ability of the teacher models. The process of knowledge distillation is shown in Figure 6.

After the knowledge distillation process is completed, hypothesis testing will be conducted to examine whether there is a statistically significant difference in performance between the teacher model and the student model, as shown in Equation (7). The model performance is evaluated based on accuracy. At a 95% confidence level, the significance level (α) is set to 0.05. Since a two-tailed test is used, if the *p*-value is less than half of the α value (i.e., 0.025), the alternative hypothesis (*H*_1_) is accepted, indicating that there is a statistically significant difference between the mean accuracy of the student model and that of the teacher model, suggesting a dissimilar performance. Conversely, if the *p*-value is greater than 0.025, the null hypothesis (*H*_0_) cannot be rejected, implying that the mean accuracies of the teacher and student models do not differ significantly, and their performance is statistically comparable. Hypothesis testing thus serves to evaluate whether the student model, after knowledge distillation, achieves a level of performance comparable to that of the teacher model.

(7) $$\left\{\begin{array}{c} H_{0}:\;\mu_{student}=\mu_{teacher}\\ H_{1}:\;\mu_{student}\neq \mu_{teacher} \end{array}\right.$$

### 3.4. Model Evaluation Metrics

To effectively evaluate model performance, this study adopts accuracy, precision, recall, and the area under the receiver operating characteristic curve (AUC) as evaluation metrics. These metrics are defined based on the four classification outcomes of the model—True Positive (*TP*), False Positive (*FP*), True Negative (*TN*), and False Negative (*FN*). The formulas for calculating accuracy, precision, recall, and AUC are defined as follows:

1. Accuracy

(8) $$Accuracy=\frac{TP+TN}{TP+FP+TN+FN}$$

2. Precision

(9) $$Precision=\frac{TP}{TP+FP}$$

3. Recall

(10) $$Recall=\frac{TP}{TP+FN}$$

4. AUC

AUC (Area Under the Curve) represents the area under the receiver operating characteristic (ROC) curve, which plots the True Positive Rate (TPR) against the False Positive Rate (*FPR*) to evaluate a model’s classification performance [51]. The formulas for FPR and *TPR* are shown in Equation (11). The value of AUC ranges from 0 to 1, with higher values indicating a better predictive performance.

(11) $$FPR=\frac{FP}{FP+TN},TPR=\frac{TP}{TP+FN}$$

### 3.5. SSO-Optimized Model

This study selects the student model with the highest accuracy and the performance that is most similar to its corresponding teacher model—among nine different teacher–student knowledge distillation combinations—as the lightweight ensemble classification model. The parameter weights of the CNN component within this selected lightweight model, obtained after completing the knowledge distillation training, are preserved and treated as a pretrained model. Subsequently, Simplified Swarm Optimization (SSO) is applied to optimize the network structure of the lightweight ensemble classification model in order to enhance its predictive performance.

**Figure 7:**
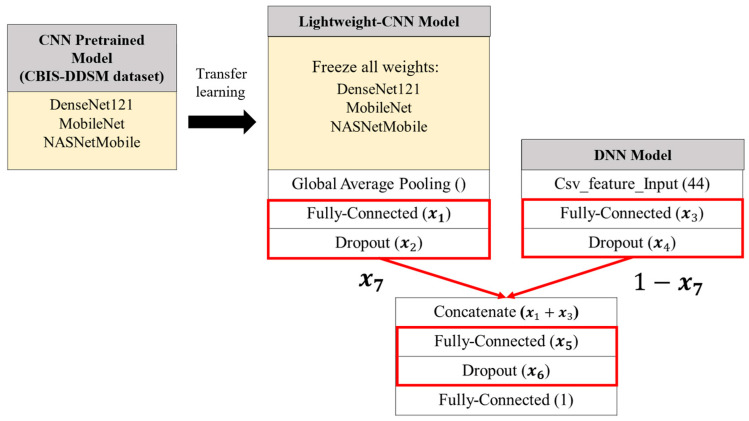
SSO-optimized architecture of the lightweight ensemble classification model.

Since the CNN pretrained model used at this stage is trained on the CBIS-DDSM dataset, all weights within the pretrained model are frozen. SSO is then employed to optimize the CNN, DNN, and the combined network structure within the lightweight ensemble model. Each of these components includes two tunable hyperparameters—the number of neurons in the fully connected layers and the dropout probability that controls the random deactivation of neurons. Additionally, the fusion ratio between image-based and tabular features is also subject to optimization. In total, seven hyperparameters are optimized to construct the final optimized lightweight ensemble classification model, as illustrated in Figure 7.

The following sections detail the encoding/decoding scheme used in SSO, the fitness function, termination criteria, update mechanism, and update procedures.

#### 3.5.1. Encoding and Decoding Method, Fitness Function Value, and Termination Criteria

**Figure 8:**
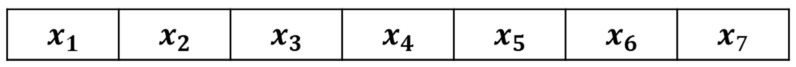
Illustration of the encoding and decoding scheme used in this study.

Using SSO (Swarm Search Optimization), the seven hyperparameters to be optimized are represented as a solution consisting of seven variables, each corresponding to a distinct hyperparameter. The encoding and decoding method used in this study is illustrated in Figure 8 and Table 5. During each SSO iteration, *N_sol_* candidate solutions are generated, each composed of seven variables, resulting in a seven-dimensional solution space.

The upper and lower bounds are defined for each variable, as follows:

- *x*_1_, *x*_3_, and *x*_5_ represent the number of neurons in fully connected layers, with a range of [1, 512]. A higher number of neurons increases the model’s capacity to fit data but also raises computational cost and overfitting risk.
- *x*_2_, *x*_4_, and *x*_6_ denote the dropout probabilities in corresponding layers, with a range of [1, 99] (%). Dropout helps prevent overfitting by randomly deactivating a proportion of neurons and their connections.
- *x*_7_ is a value between 0 and 1 that determines the fusion ratio of image features, while 1 − *x*_7_ determines the ratio for tabular features. A higher *x*_7_ value indicates a greater reliance on image features, reflecting the model’s preference for learning from them, and vice versa.

In this study on breast cancer tumor classification, the fitness function value of the SSO (Swarm Search Optimization) algorithm is computed as shown in Equation (12). Based on the design of the fitness function, the problem is formulated as a maximization problem. Initially, the seven variables are randomly initialized, and the fitness value of the initial solution is computed to obtain the initial global best solution, denoted as *gbest*. The algorithm then proceeds with iterative updates to search for the optimal solution. To enhance the efficiency of breast cancer tumor classification, the maximum number of iterations, *N_gen_*, is set as the termination criterion for the SSO algorithm. Once the number of iterations exceeds the predefined maximum, the optimization process is terminated.

*Fitness* (*X*) = *Accuracy* (12)

#### 3.5.2. Variable Definitions and Update Procedures

This study adopts the full-variable update mechanism of the SSO algorithm. In each iteration, for every solution, all variables are updated before computing the fitness function value. The updated solution is then compared with the current *gbest* and *pbest*, followed by updates to *gbest* and *pbest* as necessary. The variable definitions for the update procedure are presented in Table 6.

The SSO pseudocode is presented as Algorithm 1:
**Algorithm 1.** Pseudocode for Simplified Swarm OptimizationInput: $C_{w},C_{p},C_{g},N_{gen},N_{sol},N_{var},t,i,j,gbest=0\;(Accuracy)$
Output: gbest,G
STEP 1.For i=1 to $N_{sol}$ doSTEP 2.       Initialize $x_{i}^{0}$ randomly and calculate $F\left(x_{i}^{0}\right)$
STEP 3.       Let ${pbest}_{i}^{0}=F\left(x_{i}^{0}\right),\;P_{i}=\left(x_{i1}^{0},x_{i2}^{0},\ldots {,x}_{iNvar}^{0}\right)$
STEP 4.       If $F\left(x_{i}^{0}\right)>gbest$, then $gbest=F\left(x_{i}^{0}\right),\;G=\left(x_{i1}^{0},x_{i2}^{0},\ldots {,x}_{iNvar}^{0}\right)$
STEP 5.End ForSTEP 6.For t=1 to $N_{gen}$ and i=1 to $N_{sol}$ doSTEP 7.       For j=1 to $N_{var}$ doSTEP 8.              Update $x_{i,j}^{t}$ using Update Mechanism (UM) in Equations (2)–(9)STEP 9.       End ForSTEP 10.       If $F\left(x_{i}^{t}\right)>{pbest}_{i}^{t}$, then ${pbest}_{i}^{t}=F\left(x_{i}^{t}\right),P_{i}=\left(x_{i1}^{t},x_{i2}^{t},\ldots {,x}_{iNvar}^{t}\right)$
STEP 11.       Else go to STEP 6.STEP 12.       If $F\left(x_{i}^{t}\right)>gbest$, then $gbest=F\left(x_{i}^{t}\right),G=\left(x_{i1}^{t},x_{i2}^{t},\ldots {,x}_{iNvar}^{t}\right)$
STEP 13.  End ForSTEP 14.  Return gbest, G


## 4. Experimental Results and Analysis

This section primarily presents the experimental results and analysis. Section 4.1 describes the setup of the experimental environment. Section 4.2 analyzes the performance of the integrated classification model and two sub-models (CNN and DNN) before and after data augmentation. Section 4.3 presents the results of nine knowledge distillation configurations and hypothesis testing, ultimately identifying the best-performing student model as the lightweight integrated classification model. Section 4.4 details the experimental design for the SSO parameters, and Section 4.5 analyzes the final results of optimizing the lightweight integrated classification model using SSO.

### 4.1. The Setup of the Experimental Environment

The experimental environment used in this study consisted of a Windows 10 operating system, an Intel^®^ Core™ i5-10300H CPU (Santa Clara, CA, USA), an NVIDIA GeForce RTX 2060 GPU (Santa Clara, CA, USA), and 24 GB of RAM. All related code was implemented using Python 3.13.4.

### 4.2. The Performance of the Integrated Classification Model

To evaluate the performance of the proposed integrated classification model, the model’s hyperparameter configurations are listed in Table 7. The analysis focuses on the performance of the integrated classification model and its two sub-models—a DNN model that takes tabular data as an input and a CNN model that processes ROI images. Since the primary difference among the integrated classification models lies in the use of different CNN architectures—while the DNN architecture remains consistent—a total of 13 models are considered—6 distinct CNN models, 6 corresponding integrated classification models, and 1 standalone DNN model. This study further analyzes model performance under conditions with and without data augmentation, as well as under different data preprocessing strategies.

#### 4.2.1. Before Data Augmentation

Before data augmentation, in addition to the original ROI images, the model performance under four different image preprocessing methods will also be analyzed, according to (1) white border removal, (2) white border removal with NLM, (3) white border removal with CLAHE, and (4) white border removal with both NLM and CLAHE.

**Figure 9:**
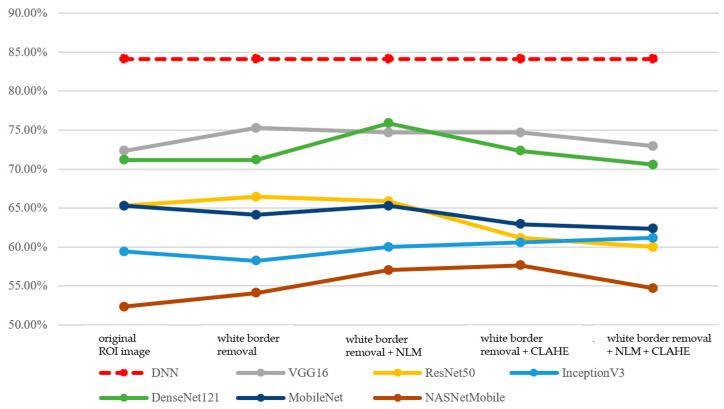
Performance of DNN and CNN models (accuracy, Before Data Augmentation).

First, the performance of the integrated classification model and its two sub-models—the CNN model and the DNN model—is analyzed. In terms of accuracy, the DNN model outperforms the CNN model, as shown in Figure 9. This may be attributed to the low color saturation and contrast of mammographic images, which makes it more challenging for models to extract distinctive features from pixel-level information. As a result, the DNN model is easier to train and demonstrates relatively better performance. In contrast, the CNN model exhibits greater performance variability due to differences in model scale and the number of parameters.

**Figure 10:**
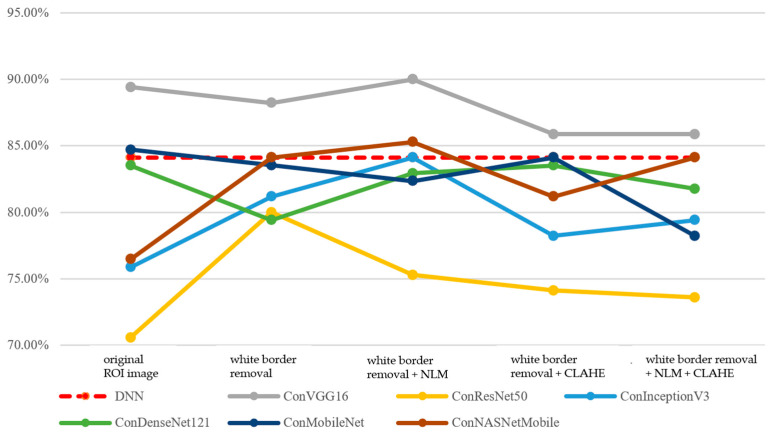
Performance of DNN and integrated classification models (accuracy, Before Data Augmentation).

Moreover, the integrated classification model, which combines features from both image and tabular data, is more susceptible to the interactions between features. As a result, some models achieve a higher accuracy than the DNN model, while others fall between the performance of the CNN and DNN models, as shown in Figure 10.

Next, the performance of the models is analyzed for the original ROI images and four different image preprocessing methods. Since the focus is on image preprocessing, the analysis primarily examines the average performance of six CNN models and six integrated classification models under different image preprocessing conditions. The performance metrics include accuracy, precision, recall, and AUC.

Firstly, in terms of accuracy, the model performance is shown in Table 8. Under the four image preprocessing conditions, both the best performance of the CNN models and the average performance of the integrated classification models were higher than those for the original ROI images. Specifically, under the image preprocessing method of white border removal with NLM, both the best and average performance of the CNN and integrated classification models exceeded those of the original ROI images, indicating that appropriate image preprocessing can enhance model accuracy.

Secondly, in terms of precision, the model performance is presented in Table 9. Among the four image preprocessing methods, the best and average performance of the CNN models—as well as the average performance of the integrated classification models—under the conditions of white border removal, white border removal with NLM, and white border removal with CLAHE all exceeded those based on the original ROI images. This indicates that in most cases, image preprocessing can improve the average precision of the models.

Next, in terms of recall, the model performance is shown in Table 10. Under all four image preprocessing methods, the best performance of the CNN models was higher than that based on the original ROI images. However, the integrated classification models showed a higher average recall only under the preprocessing methods of white border removal and white border removal with NLM. This suggests that only a limited number of image preprocessing techniques are effective in improving model recall.

Finally, in terms of AUC, the model performance is presented in Table 11. Under all four image preprocessing methods, the best performance of the CNN models exceeded that of the original ROI images. Specifically, for the preprocessing methods involving white border removal, white border removal with NLM, and white border removal with CLAHE, both the best and average performance of the CNN models and integrated classification models were higher than those based on the original ROI images. This indicates that most image preprocessing techniques can enhance the AUC of the models.

Since the dataset used in this study is balanced—that is, the number of benign and malignant cases is approximately equal—all four evaluation metrics mentioned in Section 3.4 can effectively assess the classification performance of the models. By integrating the four evaluation metrics with equal weighting, the average performance of 13 models—including 6 integrated classification models, 6 CNN models, and 1 DNN model—was analyzed across the original ROI images and four different image preprocessing methods. The results are shown in Table 12, where Δ indicates the performance difference between each preprocessing method and the original ROI images. The table shows that the preprocessing method of white border removal with NLM yields the best average performance, followed by white border removal alone. Compared to the original ROI images, the average overall performance of models improved by 1.40% with white border removal and NLM, and by 0.89% with white border removal alone. As these two preprocessing methods result in an average improvement of approximately 1% to 1.5%, they will be adopted in the subsequent analysis of model performance under data augmentation.

#### 4.2.2. After Data Augmentation

Due to the more complex architecture of convolutional neural networks (CNNs) compared to deep neural networks (DNNs), CNNs typically contain a larger number of parameters and are therefore more prone to overfitting, especially when the amount of training images is limited. To enhance the performance of the CNN model, data augmentation was applied to the training dataset using the built-in Python package Augmentor, in order to increase both the quantity and diversity of the images [46,47]. The training images were augmented through horizontal flipping, vertical flipping, and random 90-degree rotations, resulting in a four-fold increase in image quantity. A total of 5424 images were generated, which helped improve the model’s generalization ability and reduce the risk of overfitting.

**Figure 11:**
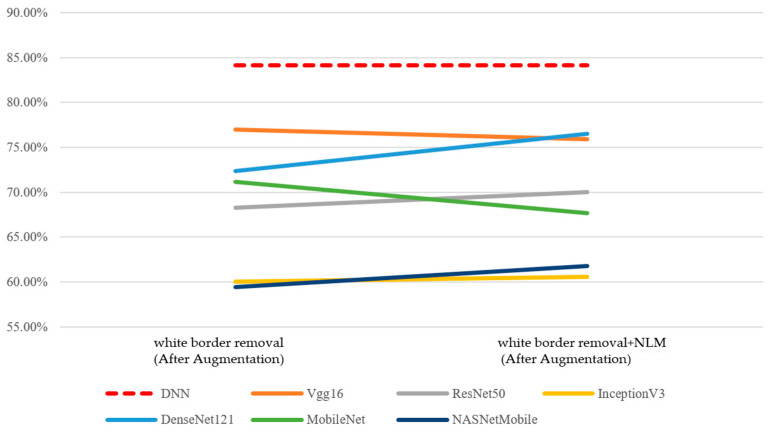
Performance of the DNN and CNN models (accuracy, After Data Augmentation).

First, the performance of the concatenated classification model and the two individual sub-models—the CNN model and the DNN model—was analyzed in Figure 11. Since preprocessing and augmentation were applied to image data, the accuracy of the DNN model remained at 84.12%. The performance of all six CNN models and six concatenated classification models showed improvement. Under the effects of data augmentation and image preprocessing, the concatenated classification models demonstrated a superior performance compared to both the standalone CNN and DNN models.

**Figure 12:**
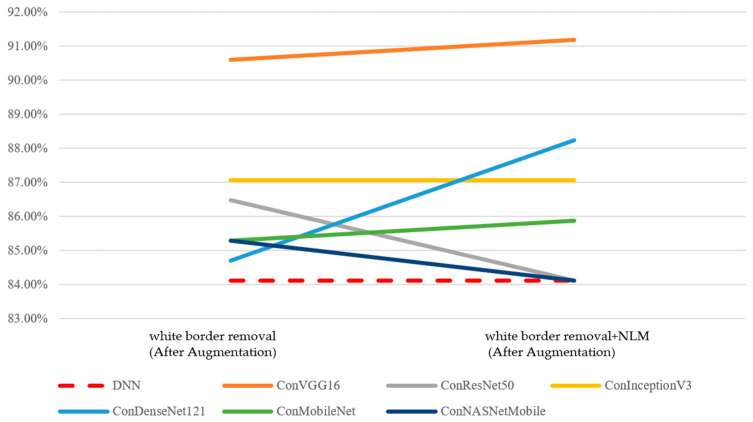
Performance of the DNN and integrated classification models (accuracy, After Data Augmentation).

Subsequently, following data augmentation, model performance was analyzed based on two image preprocessing methods—(1) the removal of white borders and (2) the removal of white borders combined with non-local mean (NLM) denoising. In terms of accuracy, the results are presented in Table 13. Compared to the original ROI images prior to augmentation, both the best and average performance of the CNN models and the concatenated classification models improved under the two preprocessing approaches. These findings indicate that image preprocessing, in conjunction with data augmentation, can further enhance model accuracy (Figure 12).

Next, in terms of precision, the model performance is shown in Table 14. Compared to the original ROI images before data augmentation, both the best and average precision of the CNN models and the concatenated classification models improved under the two image preprocessing methods. This demonstrates that image preprocessing, combined with data augmentation, can further enhance the precision of the models.

Next, in terms of recall, the model performance is presented in Table 15. Compared to the original ROI images prior to data augmentation, the best performance of the CNN models and the average performance of the concatenated classification models improved under both image preprocessing methods. However, unlike accuracy and precision, which consistently outperformed the original ROI images across all conditions, recall did not show consistent improvement in every case.

Finally, in terms of AUC, the model performance is shown in Table 16. Compared to the original ROI images before data augmentation, both the best and average AUC of the CNN models and the concatenated classification models improved under the two image preprocessing methods. This indicates that image preprocessing, in combination with data augmentation, can further enhance the models’ AUC performance.

After data augmentation, the overall average performance of the 13 models—including 6 concatenated classification models, 6 CNN models, and 1 DNN model—under the two aforementioned image preprocessing methods is summarized in Table 17. The symbol Δ represents the performance difference before and after data augmentation for each preprocessing method. As shown in the table, the average overall model performance improved by 3.4% with the removal of white borders combined with NLM, and by 2.77% with the removal of white borders alone, indicating that data augmentation contributes to enhancing average model performance.

Furthermore, as shown in Table 18, in terms of accuracy, after data augmentation and the removal of white borders as the image preprocessing method, the teacher model exhibited a higher average performance. Therefore, the removal of white borders will be adopted for the subsequent analysis of the performance of the concatenated classification models in the knowledge distillation ensemble.

### 4.3. Knowledge Distillation Experiments

After applying image preprocessing to remove white borders and performing data augmentation, three large-scale concatenated classification models—ConVGG16, ConResNet50, and ConInceptionV3—are employed as teacher models in the knowledge distillation process. Meanwhile, three small-scale concatenated classification models—ConDenseNet121, ConMobileNet, and ConNASNetMobile—are used as student models. A total of nine knowledge distillation experiments are conducted between each teacher–student pair. Following the distillation, hypothesis testing is conducted to determine whether the student models achieve a performance comparable to that of the teacher models. Specifically, a *t*-test is used to examine whether there is a statistically significant difference in accuracy between the teacher and student models. If no significant difference is observed, it can be concluded that the student models exhibit a performance similar to that of the teacher models.

#### 4.3.1. Knowledge Distillation Results

Table 19 presents the results of the nine knowledge distillation experiments, with model performance reported in terms of accuracy. The symbol Δ% indicates the change in student model accuracy before and after knowledge distillation. On average, the student models achieved a 2.52% increase in accuracy across the nine experiments. Among the three distillation pairs where ConVGG16 served as the teacher model, the student models exhibited the most notable improvements, with an average accuracy gain of approximately 4.7%. In particular, the ConNASNetMobile student model demonstrated the most significant improvement, achieving a 7.06% increase in accuracy.

#### 4.3.2. Hypothesis Testing

To evaluate whether the performance of the student model after knowledge distillation is comparable to that of the teacher model, a paired *t*-test will be conducted to examine whether there is a significant difference in the mean accuracy between the two models. At a 95% confidence level, if the *p*-value of the two-tailed paired *t*-test exceeds half of the significance level (α = 0.025), it indicates that there is no statistically significant difference in mean accuracy between the teacher and student models, suggesting a comparable model performance. The results of the hypothesis tests for the nine knowledge distillation scenarios are presented in Table 20.

Among the nine knowledge distillation configurations, six student models demonstrated accuracy levels comparable to those of their corresponding teacher models after distillation, while the remaining three did not. Notably, ConDenseNet121 achieved a performance comparable to that of all three teacher models, followed by ConNASNetMobile, which showed a comparable performance with two teacher models. ConMobileNet, on the other hand, demonstrated a comparable performance with only one teacher model. Overall, the learning effectiveness of the three student models, ranked from highest to lowest, is as follows: ConDenseNet121, ConNASNetMobile, and ConMobileNet.

#### 4.3.3. Lightweight Integrated Classification Model

**Figure 13:**
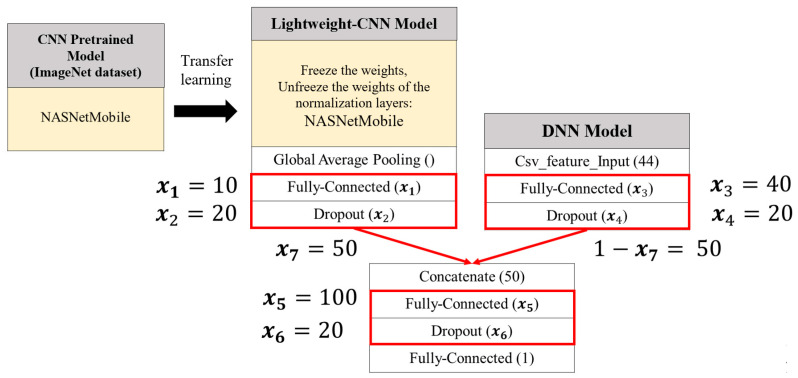
Lightweight integrated classification model.

Based on the results of knowledge distillation and hypothesis testing presented in Section 4.3.1 and Section 4.3.2, although ConNASNetMobile does not exhibit the highest learning rate, it demonstrates the most significant performance improvement through knowledge distillation. Moreover, hypothesis testing confirms that its post-distillation performance is comparable to that of the teacher model. In addition, the number of model parameters can be substantially reduced, making it well suited as a lightweight classification model for integration. Its architecture is illustrated in Figure 13. To identify the optimal model configuration, this study will proceed to the next stage by employing SSO to fine-tune the hyperparameters within the model structure.

### 4.4. SSO Experimental Design

This study retains the parameter weights of the lightweight concatenated classification model—ConNASNetMobile—after knowledge distillation training, using them as the initial weights for the SSO-optimized model, termed SSO-Concatenated NASNetMobile (SSO-CNNM). However, since each solution in SSO represents a distinct model architecture, only the weights of the CNN component of ConNASNetMobile prior to the global average pooling layer are extracted and frozen. These weights are then transferred to serve as the initial weights of the CNN component in SSO-CNNM through transfer learning. Furthermore, to achieve an improved model performance, the fitness values under different parameter allocation ratios will be evaluated, and the optimal hyperparameter combination for SSO will be selected accordingly.

#### 4.4.1. Hyperparameter Combination

Since *C_g_*, *C_p_*, and *C_w_* are probability values that increase incrementally between 0 and 1, different combinations of *C_g_*, *C_p_*, and *C_w_* create four distinct probability intervals. These intervals are defined as follows: from 0 to *C_g_* to *C_p_* to *C_w_*, and from *C_w_* to 1. These correspond to the probabilities of updating a solution to become the global best (*gbest*), personal best (*pbest*), current solution $x_{i,j}^{t}$, and a new random solution, respectively. In this study, the four probability intervals generated by *C_g_*, *C_p_*, and *C_w_* are cyclically divided according to a 7:1:1:1 ratio, such that each parameter associated with a given interval takes turns being assigned the largest proportion. This procedure results in four sets of hyperparameter combinations, with each set representing a different experimental level. The goal is to identify the influence of each factor in determining the optimal hyperparameter combination, as shown in Table 21.

To obtain more robust results, a small-sample test and one-way analysis of variance (ANOVA) will be conducted on the four experimental levels. Each experimental level will be tested five times, resulting in a total of 20 samples.

#### 4.4.2. Small-Sample Testing

**Figure 14:**
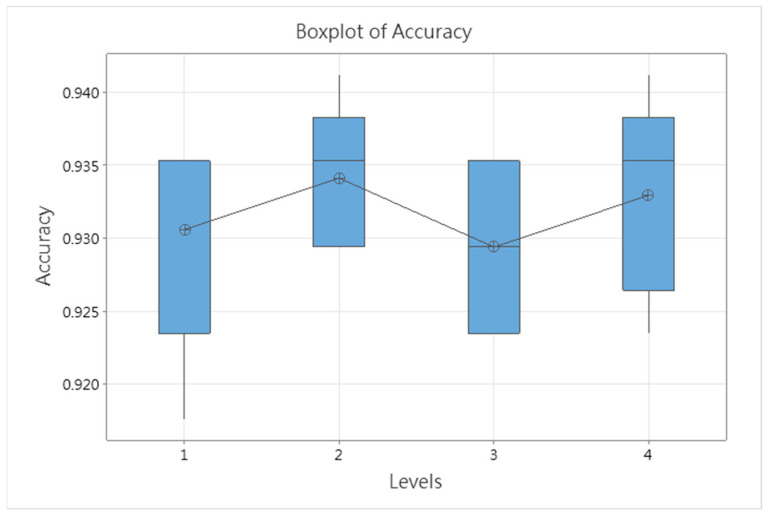
Box plot of accuracy across the four experimental levels.

Since heuristic algorithms require a significant amount of time for iterative optimization to achieve better results, this study adopts small-sample testing by conducting five experiments for each experimental level and averaging the results. In each experiment, the SSO algorithm performs 10 iterations, with 5 solutions generated per iteration (i.e., *N_gen_* = 10 and *N_sol_* = 5). The results are presented in Table 22 and Figure 14. It can be observed that when the parameter assigned the highest proportion corresponds to *pbest*, the SSO-CNNM model achieves the highest fitness function value.

#### 4.4.3. ANOVA Testing

A one-way ANOVA was conducted on the accuracy results of the four experimental levels, as shown in Table 23. The *p*-value obtained is 0.644, which is greater than the significance level α = 0.05, indicating that at the 95% confidence level, there is no statistically significant difference among the population means of the four experimental levels.

#### 4.4.4. Hyperparameter Settings

Since there is no statistically significant difference among the population means of the four experimental levels, this study selects the hyperparameter configuration based on the average fitness function values of the four levels, as shown in Table 22. Ranking the average fitness function values from highest to lowest, the corresponding dominant parameters are *pbest*, *New Random*, *gbest*, and $x_{i,j}^{t}$, respectively. Accordingly, the assigned probability values are set to 0.4 for *pbest*, 0.3 for *New Random*, 0.2 for *gbest*, and 0.1 for $x_{i,j}^{t}$. These four probability intervals are divided in a 2:4:1:3 ratio. As a result, the final SSO hyperparameters—*C_g_*, *C_p_*, and *C_w_*—are set to 0.2, 0.6, and 0.7, respectively, as shown in Table 24. Other model hyperparameters are provided in Table 7.

### 4.5. Comparison of Experimental Results

Under the hyperparameter configuration of *C_g_* = 0.2, *C_p_* = 0.6, and *C_w_* = 0.7, the SSO algorithm was executed for a total of 30 runs, with 10 iterations per run and 5 solutions generated per iteration (*N_run_* = 30, *N_gen_* = 10, and *N_sol_* = 5). For each solution, the SSO-CNNM model was trained using the following settings: 100 epochs, a batch size of 8, binary cross-entropy as the loss function, and the Adam optimizer. The results are presented in Table 25.

**Figure 15:**
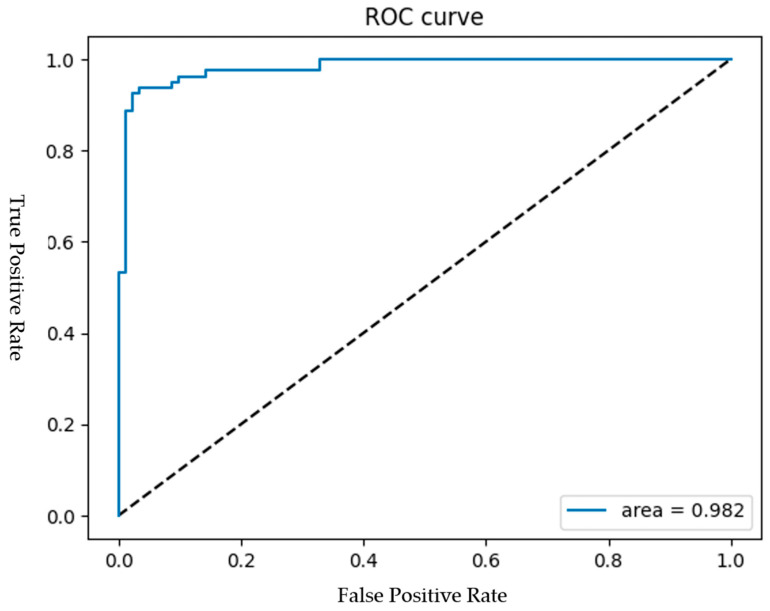
ROC curve of SSO-CNNM.

**Figure 16:**
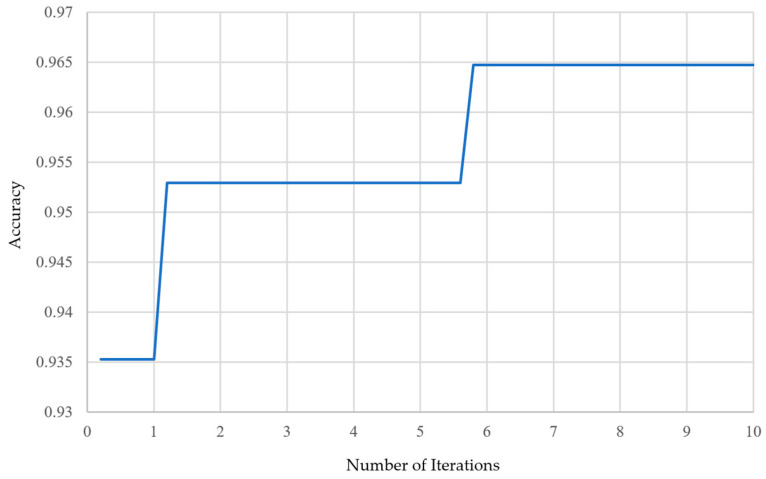
Convergence process of SSO iterative optimization.

**Figure 17:**
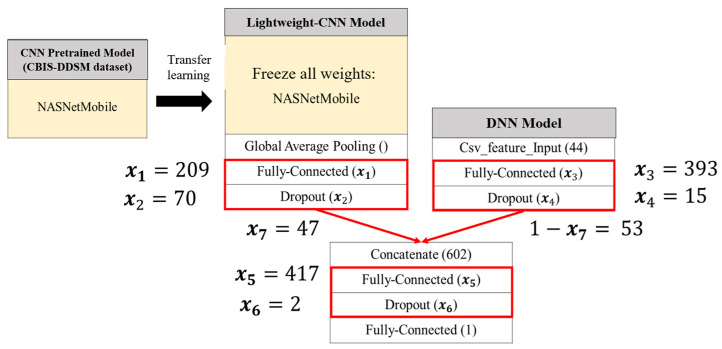
Optimized lightweight concatenated classification model (SSO-CNNM).

Among the 30 experiments, the run that achieved the best global best solution had an AUC as shown in Figure 15, and the convergence process of the SSO is illustrated in Figure 16. It can be observed that the global best solution began to converge steadily at the fourth solution in the fifth generation, reaching a value of 96.47%. The corresponding values of the seven variables are as follows: $x_{1}=209,{\;x}_{2}=70,\;x_{3}=393,{\;x}_{4}=15,x_{5}=417,{\;x}_{6}=2,{\;x}_{7}=47$. This indicates that in the CNN component of the SSO-CNNM model, the number of neurons in the fully connected layer after the global average pooling layer is 209, and the dropout rate is 70%. In the DNN component, the fully connected layer has 393 neurons and a dropout rate of 15%. After feature fusion, the fully connected layer has 417 neurons with a drt opout rate of 2%. Furthermore, the optimal fitness function value was achieved when the ratio of image features to tabular features was 47:53. The model architecture is illustrated in Figure 17.

The optimized lightweight concatenated classification model—SSO-CNNM—compared to the pre-optimization lightweight concatenated classification model—ConNASNetMobile—as shown in Figure 4, Figure 5 and Figure 6, has the following seven hyperparameters: ${(x}_{1},{\;x}_{2},\;x_{3},{\;x}_{4},x_{5},{\;x}_{6},{\;x}_{7})=(10,\;20,\;40,20,100,20,\;50)$. In this concatenated classification model, the CNN component has 10 neurons in the fully connected layer after the global average pooling layer, with a dropout rate of 20%. The DNN component has 40 neurons in the fully connected layer, with a dropout rate of 20%. After feature fusion, the fully connected layer has 100 neurons, with a dropout rate of 20%. Additionally, the weight ratio of image features to tabular features is 50:50. The following comparisons can be made.

First, in the CNN model, the number of neurons in the fully connected layer and the dropout rate are considered. The variable $x_{1}=209$ indicates that SSO increases the number of neurons to extract more features from the images. The variable $x_{2}=70$ corresponds to an increased probability of randomly shutting down neurons, which helps prevent the overfitting of the model. Overall, the optimized CNN model has a larger number of parameters compared to the pre-optimization model.

Next, in the DNN model, the number of neurons in the fully connected layer and the dropout rate are considered. The variables $x_{3}=393$ and $x_{4}=15$ indicate that due to the significant difference in the number of parameter weights between the DNN model and the pretrained CNN model, the global best solution found by SSO increases the number of neurons and reduces the probability of randomly shutting down neurons to enhance the number of parameters in the DNN model.

Next, the weight ratio between image features and tabular features is considered. The variable $x_{7}=47$ indicates that SSO assigns a nearly equal weight ratio between image features and tabular features, ensuring that both feature types are of similar importance.

Finally, the structure after combining image features and tabular features, including the number of neurons in the fully connected layer and the dropout rate, is considered. The variable $x_{5}=417$ indicates that SSO maintains a similar number of neurons in the fully connected layer after combining the CNN and DNN models, compared to the number before the combination. The variable $x_{6}=2$ shows that after applying dropout layers in both the CNN and DNN models to prevent overfitting, SSO only randomly shuts down a small number of neurons. The global best solution found by SSO results in a 4.12% improvement in model accuracy, compared to the lightweight concatenated classification model obtained through knowledge distillation.

The model performance of SSO-CNNM is shown in Table 26, where the number of parameters is referenced from the CNN model information provided in the Keras API documentation. The accuracy, precision, recall, and AUC of the SSO-CNNM model proposed in this study are 96.47%, 97.4%, 94.94%, and 98.23%, respectively. Compared to the teacher model VGG16, the parameter reduction rate is approximately 96.17%. As shown in the table, the proposed model outperforms those mentioned in the literature review, exhibiting a better performance under the same deep learning model architecture and dataset, while also achieving a significant reduction in model size.

## 5. Conclusions

This study addresses the problem of abnormal breast tumor classification. While many previous studies have employed large-capacity models to process and classify mammographic images in order to achieve a higher performance, there has been limited investigation into model lightweighting and the use of heuristic algorithms for optimizing model architectures. Therefore, this study utilizes the CBIS-DDSM dataset and applies knowledge distillation to enable three smaller-scale student models to learn from three larger-scale teacher models. The results indicate that knowledge distillation can enhance the performance of student models, with an average accuracy improvement of 2.52%. Furthermore, two-tailed hypothesis testing at a 95% confidence level confirms that after distillation, some student models achieve a performance comparable to that of their teacher models. Among them, ConNASNetMobile—showing both a significant performance improvement and comparable results to its teacher models—is selected as the lightweight classification model for integration. Its parameter weights are retained as a pretrained model, and the model architecture is further tuned and optimized using the SSO algorithm.

SSO exhibits iterative updating, rapid convergence, and strong global search capabilities. Under the selection of optimal hyperparameter combinations, the experimental results demonstrate that the global optimum identified by SSO—achieved by tuning the weight ratio between image and tabular features, the number of neurons in the fully connected layer, and the dropout rate—enables the model to effectively learn from both image and tabular features within the optimized network architecture. As a result, the model is able to identify key features and achieve an optimal classification performance.

For the integrated classification model proposed in this study, the optimal performance was achieved through a two-stage approach involving model lightweighting and SSO-based optimization. The model attained an accuracy of 96.47%, a precision of 97.4%, a recall of 94.94%, and an AUC of 98.23%, with a parameter reduction rate of 96.17%. These results demonstrate that knowledge distillation not only facilitates the construction of lightweight models but also enhances their performance. Furthermore, by optimizing the architecture of the lightweight integrated classification model using SSO, the fitness function was able to effectively escape local optima and converge within a short time to a high-quality, acceptable and feasible solution.

Traditional machine learning techniques remain effective with satisfying accuracy in breast cancer classification when combined with radiomics. While traditional machine learning models offer advantages in interpretability and compatibility with small datasets, our lightweight integrated classification model produces a better performance. This performance gap is likely due to the limited representational capacity of conventional models, which rely on handcrafted features and are sensitive to feature selection and preprocessing. However, such models remain useful in low-resource settings, when annotated data are scarce or when model explainability is prioritized, making them complementary tools rather than direct competitors in clinical workflows. In contrast, our lightweight model achieves state-of-the-art accuracy while maintaining a low computational cost, offering a practical solution for real-world clinical applications.

In future research, we will investigate how the fused features improve diagnostic insight—for example, whether BI-RADS and margin characteristics help the model distinguish subtle malignancies. We will also explore the practical implications of deploying the model in real-world clinical settings, such as on mobile ultrasound units or within PACS systems. In addition, future research will further investigate how many iterations are sufficient to achieve convergence, whether SSO is reproducible across multiple runs (i.e., variance in performance), and how SSO compares to Bayesian or evolutionary algorithms.

## Figures and Tables

**Table 1:** Description of tumor cases in the CBIS-DDSM dataset.

The Tabular Data	
Patient ID	
Breast density	levels (1–4)
Laterality (left or right breast)	2 categories (left, right)
Image view	2 categories (CC view, MLO view)
Abnormality ID	categories 1–7
Abnormality type	1 category (tumor abnormalities)
Mass shape	20 types
Mass margins	19 types
BI-RADS assessment	range from 0 to 5
Pathology	three classes—benign, benign without callback, and malignant
Subtlety	scale from 1 to 5
Image file path	
Cropped image file path	
ROI mask file path	

**Table 2:** Number of training, validation, and test samples.

Categories	Benign	Malignant	Benign-to-Malignant Ratio	Total
Training	729	627	0.54:0.46	1356
Validation	91	79	0.54:0.46	170
Test	92	78	0.54:0.46	170

**Table 3:** Input dimension standards of CNN pretrained models.

CNN Pretrained Models	Input Dimension Standards (*h*, *w*, *c*)
VGG16	Source Domain: (244, 244, 3)
	Target Domain: h, w ≥ 32, and c = 3
ResNet50	Source Domain: (244, 244, 3)
	Target Domain: h, w ≥ 32, and c = 3
InceptionV3	Source Domain: (299, 299, 3)
	Target Domain: h, w ≥ 75, and c = 3
DenseNet121	Source Domain: (244, 244, 3)
	Target Domain: h, w ≥ 32, and c = 3
NASNetMobile	Source Domain: (331, 331, 3)
	Target Domain: h, w ≥ 32, and c = 3
MobileNet	Source Domain: (244, 244, 3)
	Target Domain: h, w ≥ 32, and c = 3

**Table 4:** Number of parameters in the CNN pretrained model.

Model	Model Size	Number of Parameters	Depth
VGG16	528 MB	138.4 M	16
ResNet50	98 MB	25.6 M	107
InceptionV3	92 MB	23.9 M	189
DenseNet121	33 MB	8.1 M	242
NASNetMobile	23 MB	5.3 M	389
MobileNet	16 MB	4.3 M	55

**Table 5:** Hyperparameters and their corresponding ranges represented by the SSO solution variables.

Solution Variables	Hyperparameters	Ranges	Unit
*x*_1_	Number of neurons in the fully connected layers of the CNN model	[1, 512]	number
*x*_2_	Dropout rate in the CNN model (probability of randomly deactivating neurons)	[1, 99]	%
*x*_3_	Number of neurons in the fully connected layers of the DNN model	[1, 512]	number
*x*_4_	Dropout rate in the DNN model (probability of randomly deactivating neurons)	[1, 99]	%
*x*_5_	Number of neurons in the fully connected layers of the concatenated classification model	[1, 512]	number
*x*_6_	Dropout rate in the concatenated classification model (probability of randomly deactivating neurons)	[1, 99]	%
*x*_7_	Fusion ratio between image features and tabular features	[1, 99]	%

**Table 6:** SSO variable descriptions.

Variable	Definition
*N_var_*	Represents the number of variables in a solution
*N_sol_*	Represents the number of solutions in a single iteration
*N_gen_*	Represents the number of iterations in the SSO algorithm
*N_run_*	Represents the number of experimental runs of the SSO algorithm
*t*	Records the current execution status of *N_gen_*, *t* =1, 2, …, *N_gen_*
*i*	Records the current execution status of *N_sol_*, *t* =1, 2, …, *N_sol_*
*j*	Records the current execution status of *N_var_*, *t* =1, 2, …, *N_var_*
$x_{i,j}^{t}$	Represents the value of the *j*-th variable in the *i*-th solution at iteration *t*
$x_{i}^{t}$	$x_{i}^{t}=\left(x_{i1}^{t},x_{i2}^{t},\ldots x_{iNvar}^{t}\right)$ Records all variable values of the *i*-th solution at iteration *t*
$F\left(x_{i}^{t}\right)$	Fitness function value of the *i*-th solution at iteration *t*
*gbest*	Represents the fitness function value of the global best solution
${pbest}_{i}^{t}$	Represents the fitness function value of the local best solution of the *i*-th solution at iteration *t*
*G*	$G=\left(x_{i1}^{t},x_{i2}^{t},\ldots {,x}_{iNvar}^{t}\right)$ Records all variable values of the global best solution
*P_i_*	$P_{i}=\left(p_{i,1},p_{i,2},\ldots ,p_{i,N_{var}}\right)$ Records all variable values of the local best solution of the *i*-th solution
$\rho_{i}^{t}$	$\rho_{i}^{t}=\left(\rho_{i1}^{t},\rho_{i2}^{t},\ldots ,\rho_{iNvar}^{t}\right)$ Represents a set of uniformly distributed random numbers in the range [0, 1], used to determine the update method of the *i*-th solution’s variables
*C_g_*, *C_p_*, *C_w_*	Represents the upper and lower bounds of different update intervals

**Table 7:** Hyperparameter configuration of the lightweight integrated classification model.

Hyperparameter	Value
Batch size	8
Epoch	100
Early Stopping	monitor = ‘val_loss’, patience = 4
Learning rate	1 × 10^−3^
ReduceLROnPlateau	monitor = ‘val_loss’, factor = 0.2, patience = 2, min_lr = ${1e}^{-4}$
Dropout	0.2
Optimizer	Adam
Loss function	Binary cross-entropy

**Table 8:** The model accuracy of four image preprocessing methods.

Accuracy				
Image Preprocessing	CNN Model		Integrated Classification Model	
	Best Performance	Average Performance	Best Performance	Average Performance
Original ROI image	72.35%	64.31%	89.41%	80.10%
White border removal	75.29%	64.90%	88.24%	82.75%
White border removal + NLM	75.88%	66.47%	90.00%	83.33%
White border removal + CLAHE	74.71%	64.90%	85.88%	81.18%
White border removal + NLM + CLAHE	72.94%	63.63%	85.88%	80.49%

**Table 9:** The model precision of four image preprocessing methods.

Precision				
Image Preprocessing	CNN Model		Integrated Classification Model	
	Best Performance	Average Performance	Best Performance	Average Performance
Original ROI image	70.13%	61.00%	86.59%	76.27%
White border removal	73.08%	61.63%	83.72%	77.63%
White border removal + NLM	74.65%	63.84%	83.54%	79.43%
White border removal + CLAHE	71.83%	61.24%	82.14%	77.47%
White border removal + NLM + CLAHE	68.60%	60.77%	81.40%	76.50%

**Table 10:** The model recall of four image preprocessing methods.

Recall				
Image Preprocessing	CNN Model		Integrated Classification Model	
	Best Performance	Average Performance	Best Performance	Average Performance
Original ROI image	71.79%	65.17%	98.72%	83.97%
White border removal	73.08%	60.04%	92.31%	87.82%
White border removal + NLM	82.05%	60.26%	97.44%	85.90%
White border removal + CLAHE	80.77%	64.10%	91.03%	83.12%
White border removal + NLM + CLAHE	75.64%	62.63%	94.87%	83.33%

**Table 11:** The model AUC of four image preprocessing methods.

AUC				
Image Preprocessing	CNN Model		Integrated Classification Model	
	Best Performance	Average Performance	Best Performance	Average Performance
Original ROI image	80.72%	68.40%	94.97%	87.51%
White border removal	81.90%	68.69%	95.99%	90.99%
White border removal + NLM	82.75%	69.13%	96.33%	90.54%
White border removal + CLAHE	81.68%	69.62%	95.21%	88.68%
White border removal + NLM + CLAHE	81.55%	67.62%	94.45%	87.38%

**Table 12:** Average overall model performance under four image preprocessing methods (unit: %).

Evaluation Metrics	Original ROI Image	White Border Removal	Δ	White Border Removal + NLM	Δ	White Border Removal + CLAHE	Δ	White Border Removal + NLM + CLAHE	Δ
Accuracy	73.12	74.62	1.49	75.61	2.49	73.89	0.77	72.99	−0.14
Precision	69.62	70.54	0.92	72.39	2.77	70.28	0.66	69.62	0.00
Recall	75.35	74.75	−0.59	73.96	−1.38	74.46	−0.89	73.88	−1.47
AUC	79.15	80.89	1.74	80.88	1.74	80.25	1.10	78.73	−0.41
Average Performance	74.31	75.20	0.89	75.71	1.40	74.72	0.40	73.80	−0.51

**Table 13:** Model accuracy under two image preprocessing methods.

Accuracy				
Image Preprocessing	CNN Model		Integrated Classification Model	
	Best Performance	Average Performance	Best Performance	Average Performance
Original ROI image	72.35%	64.31%	89.41%	80.10%
White border removal	77.06%	68.04%	90.59%	86.57%
White border removal + NLM	76.47%	68.73%	91.18%	86.76%

**Table 14:** Model precision under two image preprocessing methods.

Precision				
Image Preprocessing	CNN Model		Integrated Classification Model	
	Best Performance	Average Performance	Best Performance	Average Performance
Original ROI image	70.13%	61.00%	86.59%	76.27%
White border removal	74.07%	66.12%	86.90%	83.45%
White border removal + NLM	73.42%	65.41%	87.06%	82.38%

**Table 15:** Model recall under two image preprocessing methods.

Recall				
Image Preprocessing	CNN Model		Integrated Classification Model	
	Best Performance	Average Performance	Best Performance	Average Performance
Original ROI image	71.79%	65.17%	98.72%	83.97%
White border removal	76.92%	60.47%	93.59%	88.25%
White border removal + NLM	82.05%	67.52%	94.87%	90.60%

**Table 16:** Model AUC under two image preprocessing methods.

AUC				
Image Preprocessing	CNN Model		Integrated Classification Model	
	Best Performance	Average Performance	Best Performance	Average Performance
Original ROI image	80.72%	68.40%	94.97%	87.51%
White border removal	83.06%	72.38%	95.39%	93.19%
White border removal + NLM	82.51%	73.51%	95.96%	93.41%

**Table 17:** Average overall model performance under two image preprocessing methods (unit: %).

Evaluation Metrics	White Border Removal (Before Data Augmentation)	White Border Removal (After Data Augmentation)	Δ	White Border Removal + NLM (Before Data Augmentation)	White Border Removal + NLM (After Data Augmentation)	Δ
Accuracy	74.62	77.83	3.21	75.61	78.24	2.63
Precision	70.54	75.30	4.76	72.39	74.48	2.09
Recall	74.75	75.15	0.40	73.96	79.49	5.53
AUC	80.89	83.61	2.72	80.88	84.23	3.35
Average Performance	75.20	77.97	2.77	75.71	79.11	3.40

**Table 18:** Teacher model accuracy under two image preprocessing methods.

Integrated Classification Model	White Border Removal (After Data Augmentation)	White Border Removal + NLM (After Data Augmentation)
ConVGG16	90.59%	91.18%
ConResNet50	86.47%	84.12%
ConInceptionV3	87.06%	87.06%
Average Performance	88.04%	87.45%

**Table 19:** Model performance (accuracy) of the nine knowledge distillation experiments.

Knowledge Distillation Combination		Before Knowledge Distillation		After Knowledge Distillation	Δ%
Teacher Model	Student Model	Teacher Model	Student Model	Student Model	
ConVGG16	ConDenseNet121	90.59%	84.71%	88.82%	4.11%
	ConMobileNet		85.29%	88.24%	2.95%
	ConNASNetMobile		85.29%	92.35%	7.06%
ConResNet50	ConDenseNet121	86.47%	84.71%	85.29%	0.58%
	ConMobileNet		85.29%	86.47%	1.18%
	ConNASNetMobile		85.29%	85.88%	0.59%
ConInceptionV3	ConDenseNet121	87.06%	84.71%	88.24%	3.35%
	ConMobileNet		85.29%	86.47%	1.18%
	ConNASNetMobile		85.29%	87.01%	1.72%

**Table 20:** Hypothesis testing for the nine knowledge distillation scenarios.

Knowledge Distillation Configurations		After Knowledge Distillation	
Teacher Model	Student Model	*p*-Value	Performance of the Teacher and Student Models
ConVGG16	ConDenseNet121	0.0474 > 0.025	Comparable
	ConMobileNet	0.0002 < 0.025	Not Comparable
	ConNASNetMobile	0.0396 > 0.025	Comparable
ConResNet50	ConDenseNet121	0.3344 > 0.025	Comparable
	ConMobileNet	0.6710 > 0.025	Comparable
	ConNASNetMobile	0.9818 > 0.025	Comparable
ConInceptionV3	ConDenseNet121	0.0384 > 0.025	Comparable
	ConMobileNet	0.0019 < 0.025	Not Comparable
	ConNASNetMobile	0.0115 < 0.025	Not Comparable

**Table 21:** Four experimental levels.

Experimental Levels	$C_{g}$	$C_{p}$	$C_{w}$	gbest$:pbest:x_{i,j}^{t}$:New Random
1	0.7	0.8	0.9	7:1:1:1
2	0.1	0.8	0.9	1:7:1:1
3	0.1	0.2	0.9	1:1:7:1
4	0.1	0.2	0.3	1:1:1:7

**Table 22:** Fitness function values for different experimental levels.

Experimental Levels	${(C}_{g},C_{p},C_{w})$	Dominant Proportion Parameter	Best	Average
1	(0.7, 0.8, 0.9)	gbest	93.53%	93.01%
2	(0.1, 0.8, 0.9)	pbest	94.12%	93.41%
3	(0.1, 0.2, 0.9)	$x_{i,j}^{t}$	93.53%	92.94%
4	(0.1, 0.2, 0.3)	New Random	94.12%	93.29%

**Table 23:** One-way ANOVA test.

Source	DF	Adj SS	Adj MS	*F*-Value	*p*-Value
Level	3	0.000070	0.000023	0.57	0.644
Error	16	0.000654	0.000041		
Total	19	0.000724			

**Table 24:** Hyperparameter configurations.

Hyperparameter	$C_{g}$	$C_{p}$	$C_{w}$	$N_{run}$	$N_{gen}$	$N_{sol}$
Value	0.2	0.6	0.7	30	10	5

**Table 25:** Global best solution in SSO iterations.

	Accuracy	Precision	Recall	AUC	$x_{1}$	$x_{2}$	$x_{3}$	$x_{4}$	$x_{5}$	$x_{6}$	$x_{7}$
Best	0.9647	0.9740	0.9494	0.9823	209	70	393	15	417	2	47
Average	0.9529	0.9477	0.9515	0.9863	145.3	73.7	287.9	19.6	331.5	49.8	37.9

**Table 26:** Experimental comparison results.

Method	Benchmark Model	Number of Parameters	Accuracy	AUC
Transfer Learning and Fine-Tuning [52]	VGG16	138.4 M	84.00%	0.8440
CNN Architecture Using Depthwise Separable Convolution Layers and Normalization Layers [53]	FC-DSCNN	143.7 M	87.00%	0.8600
CNN Architecture Using Special Region Pooling Layers [54]	DenseNet169	14.3 M	76.20%	
Ensemble Model [24]	ResNet50V2, ResNet101V2, ResNet152V2	130.7 M	95.13%	0.9500
Multi-Stage CNN Feature Extraction, MEWOA Feature Selection, and Cubic SVM Classification [55]	MobileNetV2, NASNetMobile	8.8 M	93.80%	
Multi-View Feature Fusion Model [25]	DenseNet	16.2 M	95.24%	0.9503
This study—SSO-CNNM	NASNetMobile	5.3 M	96.47%	0.9823

## Data Availability

CBIS-DDSM (Curated Breast Imaging Subset of DDSM) dataset can be freely downloaded from Cancer Imaging Archive (TCIA) of National Institutes of Health (NIH) (https://www.cancerimagingarchive.net/collection/cbis-ddsm/).
